# SlHB8 Is a Novel Factor in Enhancing Cold Resistance in Tomato Anthers by Modulating Tapetal Cell Death

**DOI:** 10.3390/ijms25179336

**Published:** 2024-08-28

**Authors:** Hongling Guan, Canye Yu, Zaohai Zeng, Huimin Hu, Yuxiang Lin, Caiyu Wu, Yiwen Yao, Rui Xia, Zhengguo Li, Chongjian Ma, Riyuan Chen, Baowen Huang, Yanwei Hao

**Affiliations:** 1 Key Laboratory of Horticultural Crop Biology and Germplasm Innovation in South China, Ministry of Agriculture, College of Horticulture, South China Agricultural University, Guangzhou 510642, China; guanhl@scau.edu.cn (H.G.); cyyu@stu.scau.edu.cn (C.Y.); zengzh@scau.edu.cn (Z.Z.); huhuimin1013@163.com (H.H.); lyxtulip@163.com (Y.L.); wucaiyu1995@163.com (C.W.); prometheus03@163.com (Y.Y.); rxia@scau.edu.cn (R.X.); rychen@scau.edu.cn (R.C.); 2 Guangdong Provincial Key Laboratory of Utilization and Conservation of Food and Medicinal Resources in Northern Region, School of Biology and Agriculture, Shaoguan University, Shaoguan 512005, China; chjma@sgu.edu.cn; 3 Key Laboratory of Plant Hormones and Development Regulation of Chongqing, School of Life Sciences, Chongqing University, Chongqing 400044, China; zhengguoli@cqu.edu.cn

**Keywords:** tomato, cold stress, tapetum, programmed cell death, pollen development

## Abstract

Tomato plants favor warmth, making them particularly susceptible to cold conditions, especially their reproductive development. Therefore, understanding how pollen reacts to cold stress is vital for selecting and improving cold-resistant tomato varieties. The programmed cell death (PCD) in the tapetum is particularly susceptible to cold temperatures which could hinder the degradation of the tapetal layer in the anthers, thus affecting pollen development. However, it is not clear yet how genes integral to tapetal degradation respond to cold stress. Here, we report that *SlHB8*, working upstream of the conserved genetic module DYT1-TDF1-AMS-MYB80, is crucial for regulating cold tolerance in tomato anthers. *SlHB8* expression increases in the tapetum when exposed to low temperatures. CRISPR/Cas9-generated SlHB8-knockout mutants exhibit improved pollen cold tolerance due to the reduced temperature sensitivity of the tapetum. SlHB8 directly upregulates *SlDYT1* and *SlMYB80* by binding to their promoters. In normal anthers, cold treatment boosts *SlHB8* levels, which then elevates the expression of genes like *SlDYT1*, *SlTDF1*, *SlAMS*, and *SlMYB80*; however, *slhb8* mutants do not show this gene activation during cold stress, leading to a complete blockage of delayed tapetal programmed cell death (PCD). Furthermore, we found that SlHB8 can interact with both SlTDF1 and SlMYB80, suggesting the possibility that SlHB8 might regulate tapetal PCD at the protein level. This study sheds light on molecular mechanisms of anther adaptation to temperature fluctuations.

## 1. Introduction

The tomato (*Solanum lycopersicum*) is a major worldwide horticultural crop, with its farming and yield recently increasing in many areas [1]. Native to warm tropical and subtropical areas, tomato is susceptible to abiotic stress factors such as low temperatures. Like other crops, cold stress negatively impacts both the vegetative growth and reproductive development of tomato, with the latter being more sensitive [2,3]. Anthers are sensitive to cold stress, and pollen development is the weakest aspect of plant sexual reproduction when conditions are unfavorable [4,5]. The impact of cold stress includes a decrease in pollen vitality and quantity, abnormalities in pollen wall structure, a lack of internal pollen substances, and irregularities in tapetum degradation [6,7]. These effects can eventually result in flower drop, fruit set reduction, and yield decrement. Therefore, understanding the physiological and molecular mechanisms of plant responses to cold stress during the reproductive stage possesses substantial economic and societal value for enhancing the yield and quality of plant products under cold stress conditions [8].

C-repeat binding factors (CBFs) are key transcription factors involved in the response to cold stress [9]. Research indicates that SlPIF4 mediates the cold stress response by regulating the downstream *CBF1*. This regulation resists cold by negatively modulating gibberellin (GA) signals while positively modulating the biosynthesis and signal transduction of abscisic acid (ABA) and jasmonic acid (JA) [10]. Interestingly, genes with increased expression in seedlings or leaves related to cold stress response are not triggered, nor do they exhibit weak expression in anthers after cold stress exposure. This implies that the response mechanisms during plant seedling and anther development are not completely identical when it comes to cold stress [7,11].

The tapetum, the innermost cell layer of the anther chambers, envelops the microspores [12]. It supplies neighboring microspores with nutrients like lipids and polysaccharides during their normal development [13,14]. Tapetum degradation within anthers must occur at a specific stage of anther development to ensure normal tapetal functionality and pollen wall growth. Premature or delayed degradation of the tapetum results in male sterility in pollen [15,16,17]. For example, in plants like Arabidopsis, rice, and wheat, heat stress triggers premature tapetum degradation [18,19,20,21]. On the other hand, cold stress can inhibit tapetum development by either delaying or suppressing its PCD [22,23,24,25]. In rice, exposure to cold stress at an early pollen development stage disrupts the regular tapetum PCD process, leading to an accumulation of cellular degradation by-products from programmed death, causing tapetal cells to swell and eventually resulting in pollen sterility [26]. Although it is observed in many plants that cold stress can affect pollen development through delaying the tapetum degradation in the anther, the response mechanism of tapetum degradation genes under cold stress remains obscure.

The molecular mechanisms of anther tapetum development and degradation are regulated by a genetic module including DYT1 (DYSFUNCTIONAL TAPETUM 1), TDF1 (DEFECTIVE IN TAPETAL DEVELOPMENT AND FUNCTION 1), AMS (ABORTED MICROSPORES), and MYB80/103. This module, conserved across crops such as Arabidopsis, rice, maize, and tomato, controls both tapetum development and programmed cell death (PCD) [27,28,29,30]. Early tapetum degradation is governed by DYT, TDF1, and AMS, while MYB80 and MS1 significantly contribute to late-stage tapetum degradation, microspore release, and the transport of pollen wall precursors. Mutants of these genes (*dyt1*, *tdf1*, *ams*, and *ms1*) often show delayed tapetum degradation, while the *myb80* mutant exhibits premature degradation. Despite insights into the molecular network regulating tapetal cell PCD, relationships between this conserved module and cold stress are understudied. In tomatoes, the expression levels of the DYT1-TDF1-AMS-MYB80 components responsible for tapetum PCD increase post-cold stress [31]. The bHLH transcription factor SlPIF4, in response to cold stress, directly interacts with the tapetum PCD-regulating module to negatively correlate with anther cold tolerance. The tapetum of the *slpif4* mutant shows less sensitivity to temperature and is cold resistant. Studies have shown that SlPIF4 directly interacts with SlDYT1. Cold stress stimulates the formation of the dimer SlPIF4-SlDYT1, enhancing *SlTDF1* transcription activation, causing delayed tapetum degradation and pollen sterility. Under cold stress, *SlTDF1* transcription activation is suppressed in the *slpif4* mutant, leading to stronger pollen cold resistance, enhancing tomato fruit setting and yield [31]. Thus, manipulating tapetum PCD under cold conditions might enhance pollen vitality during cold stress.

SlHB8 is a member of the HD-zip III transcription factor family in tomatoes, which also includes SlHB15A, SlHB15B, SlREV, SlPHB, and SlPHV. These genes are all negatively regulated by *miRNA166* [32]. Our previous studies found that SlHB8 functions in many parts of tomato plants. The loss of function of *SlHB8* resulted in thicker stems and shorter plants [33]. The overexpression of *SlHB8* led to a rolling leaf and thinner stem, as well as aborted pollen [33,34,35]. Research indicates that low temperatures increase *miRNA166* levels, which modulates the expression of *SlHB15A* in a dose-dependent manner. This regulation is critical for developing seedless fruits in *PF1/pf1* tomato plants under cold stress [32], implying that these family genes contribute to the tomato’s response to cold stress. Previous studies have demonstrated that SlHB8 plays a role in tapetum development and degradation, a vital process for anther maturation by positively affecting a conserved genetic pathway involving DYT1-TDF1-AMS-MYB80 [33]. Therefore, it is plausible that SlHB8 may influence pollen development under cold conditions.

In this study, we explored how SlHB8 regulates anther adaptation to cold stress by examining CRISPR/Cas9-generated SlHB8-knockout mutants. Our findings indicate that cold treatment triggers an increased expression of *SlHB8* in the tapetum, which in turn enhances cold tolerance in *slhb8* anthers as a result of alterations in tapetal programmed cell death (PCD). In wild-type anthers, the increased *SlHB8* expression induces the *SlDYT1* transcripts, which form a dimer with SlPIF4. This dimer activates the downstream regulators *SlTDF1*, *SlAMS*, and *SlMYB80*, thereby leading to a delay in tapetum degradation and subsequent pollen abortion; however, in *slhb8* mutants, cold exposure does not result in an increase in *SlDYT1* expression, leading to an unsuccessful increase in *SlTDF1*, *SlAMS*, and *SlMYB80*. This lack of increase results in the regular tapetal PCD (Figure S4). We learned that SlHB8 can also interact with SlTDF1 and SlMYB80, suggesting the possibility that SlHB8 might regulate tapetal PCD at the protein level. In summary, our research sheds light on the molecular mechanisms that facilitate anther adaptation to temperature changes.

## 2. Results

### 2.1. SlHB8 Showed a Cold-Induced Expression Pattern within the Tapetal Cells of Tomato Anthers

**Figure 1:**
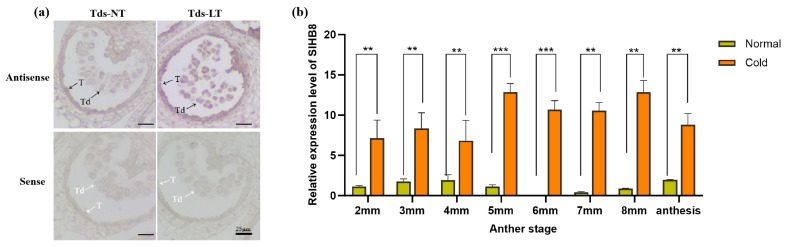
*SlHB8* expression pattern analysis under cold treatment. (**a**) *SlHB8* in situ hybridization was performed on cross-sectioned wild type anthers at the tetrad stage under normal and cold stress conditions. The upper and lower probes correspond to antisense and sense of *SlHB8*, respectively. The black and white arrows denote positive and negative in situ hybridization signals for *SlHB8* transcripts. NT: normal temperature; LT: low temperature; T: tapetum; Td: tetrad; Tds: tetrad stage. Bars = 25 μm. (**b**) qRT-PCR analysis of *SlHB8* expression in anthers at different stages under cold treatment; 2 mm: flower bud of 2 mm length; anthesis: anther at anthesis stage. Significant differences in mean values are indicated by an asterisk: ** *p*-value < 0.01, *** *p*-value < 0.001; Student’s *t*-test.

Preliminary research indicated that *SlHB8* is expressed in both the tapetum and microspores from the microsporocyte stage to the maturation of pollen grains under normal temperatures [33]. To explore how *SlHB8* expression changes in anthers under cold stress, we analyzed its spatiotemporal pattern during the tetrad stage using in situ hybridization. Our findings indicated that at normal temperatures, positive signals for the antisense probe were present throughout the anther at this stage, with stronger signals observed in the tapetum and microspores (Figure 1a). However, exposure to low temperatures resulted in significantly stronger positive signals across the anther—especially within tapetal cells and microspores (Figure 1a). Quantitative PCR results also confirmed increased levels of *SlHB8* expression in anthers during the pollen development after low-temperature treatment (Figure 1b), implying that *SlHB8* may be involved in regulating pollen development and tapetum degradation during cold stress.

### 2.2. Knocking Out SlHB8 Enhances the Tolerance of Anthers to Low-Temperature Stress

**Figure 2:**
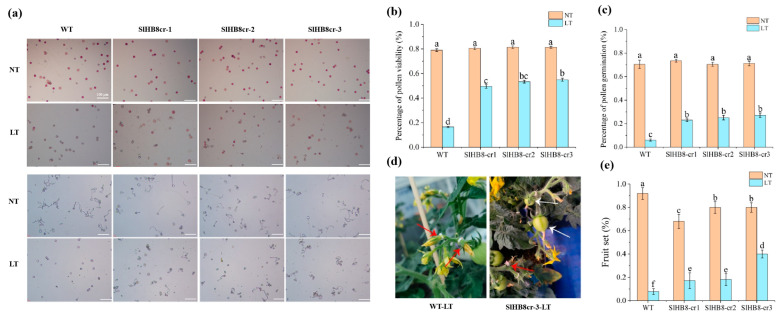
Pollen viability and pollen tube gemination of *SlHB8* gene knockout plants. Buds of 4 mm in size (tetrad stage) were labeled, subjected to cold treatment for seven days, and subsequently cultivated at normal temperature until the labeled flowers were fully open, at which point anthers were harvested for pollen viability assessment. (**a**) Pollen TTC staining (upper) and in vitro germination tests (lower) on pollen were analyzed in the wild type and *SlHB8* mutants under normal and cold conditions. Scale bars = 100 μm. (**b**) The pollen vitality of WT and *SlHB8* mutants under normal temperature and cold stress. Scale bars = 100 μm. (**c**) The pollen tube germination rate of WT and *SlHB8* mutants under normal temperature and cold stress. The data are representative of at least 30 samples, quoted as the mean ± standard deviation, with a Tukey HSD, *p*-value < 0.05. (**d**) Image depicting the fruit set of the wild type and *slhb8* after low-temperature stress at the tetrad stage. White arrows denote successful fruit sets; red arrows indicate failures. (**e**) Fruit-set rates of the wild type and *slhb8* mutants under normal and low-temperature stress. A minimum of 10 plants per genotype were evaluated. Data are reported as the mean ± standard deviation. Significant differences in mean values are indicated by the letters a–f: Tukey HSD, *p* < 0.05.

*SlHB8*, activated by cold in the tapetum, appears to play a role in pollen development under low temperatures. We examined pollen viability for both wild-type *SlHB8* and three loss of function mutants generated by using CRISPR/Cas9 technology at room temperature and during cold stress. At a standard temperature of 25 °C/20 °C, pollen viability was similar across all plants: 79.0% for the wild type (WT), 80.6% for *SlHB8cr-1*, 81.4% for *SlHB8cr-2*, and 81.3% for *SlHB8cr-3* (Figure 2a,b). However, after exposure to low conditions of 15 °C/10 °C, the WT plant pollen showed a drastic drop in viability to just 16.5%. The *SlHB8cr* mutants behaved better under these conditions with viabilities of 49.5%, 53.4%, and 54.9%, respectively, triple that of WT (Figure 2a,b). In an in vitro assay measuring pollen tube germination at room temperature, rates were comparable among all plants: WT at 70.5%, *SlHB8cr-1* at 73.4%, *SlHB8cr-2* at 70.5%, and *SlHB8cr-3* at 71.2%; no significant differences were observed (Figure 2a,c). Following cold treatment though, germination rates dropped sharply across all plants, with WT being most affected as it fell to only 6.0%. In contrast, the mutants’ germination rates remained higher: 23.0% for *SlHBcr-1*, 25.1% for *SlHBcr-2*, and 27.1% for *SlHBcr-3*, about four times greater than the rate seen in WT (Figure 2a,c).

Exposure to low temperatures during reproductive development leads to reduced pollen vitality in tomatoes, negatively impacting both fruit yield and fruit quality. Aiming to find out whether cold temperatures influence the fruit yield and quality of *SlHB8* mutants, we measured the fruit-set rates, fruit size, fruit weight, and seed number in wild-type and *SlHB8* mutant plants. The results showed that at room temperature, the wild-type plants had a 92.0% fruit-set rate, significantly higher than the mutants (Figure 2d,e). However, when under cold treatment, the wild-type’s fruit-set rate dropped to 7.7%, much lower than under normal conditions. In contrast, the *SlHB8* mutants exhibited better values than the wild type: *SlHB8cr-1* and *SlHB8cr-2* had fruit-set rates of 17.1% and 18.1%, respectively, more than double that of the wild type; and *SlHB8cr-3* reached a rate of 40.0%, over four times higher than that of the wild type (Figure 2d,e).

**Figure 3:**
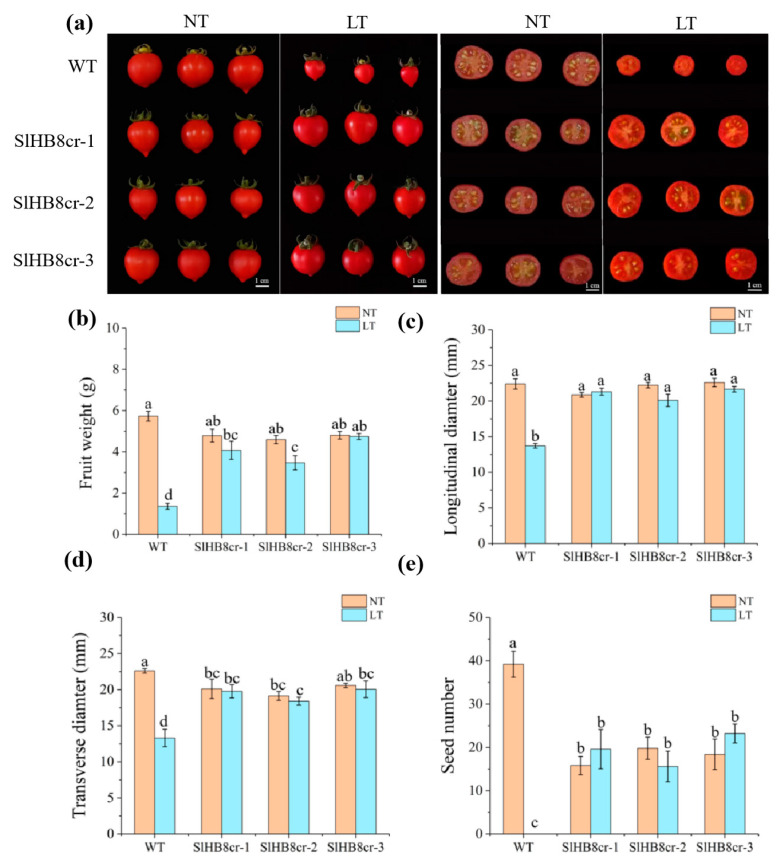
Fruit morphology analysis of the wild type and *slhb8* mutants under normal and cold conditions. (**a**) Fruit images of the wild type and *hb8* mutants under normal and low-temperature treatments; (**b**) the weight of tomato fruits from the wild type and *slhb8* mutants under normal and cold stress; the longitudinal diameter (**c**) and transverse diameter (**d**) of tomato fruits from the wild type and *slhb8* mutants under normal and cold stress; (**e**) the seed number per tomato fruit of the wild type and *slhb8* mutants under normal and cold stress. Data are expressed as the mean ± standard deviation. Significant differences in mean values are indicated by the letters a–d: Tukey HSD, *p* < 0.05.

After cold treatment, wild-type plants yielded small seedless fruits that were significantly lighter and smaller (Figure 3a–d). In contrast, the weight and size of fruits from *SlHB8* mutants were similar regardless of whether they grew in low temperatures or at room temperature (Figure 3b–d). Moreover, *SlHB8* mutants could produce several seeds under cold conditions (Figure 3e). These findings suggest that cold stress impairs pollen viability or induces sterility in wild-type plants, whereas the pollen from *SlHB8* mutants retains its biological function even under cold stress conditions.

### 2.3. SlHB8 Negatively Regulates Low Temperature Tolerance of Tomato Anthers by Regulating Tapetal PCD

**Figure 4:**
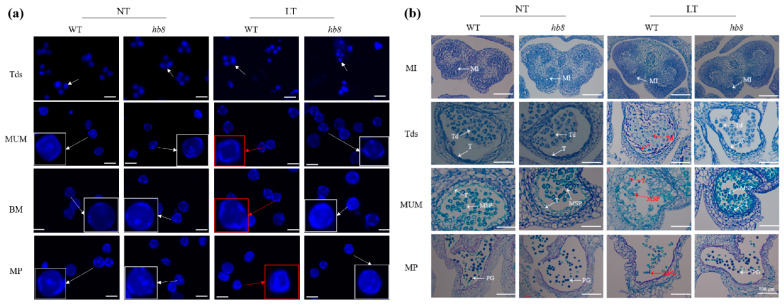
Observation of pollen and anther morphology in the wild type and *slhb8* mutant under microscopy. DAPI staining (**a**) and histocytological observation (**b**) of pollen development in the wild type and *slhb8* mutant. The scale bar in subfigure a represents 60 μm. White arrows indicate pollen with nuclei; red arrows denote anucleate pollen; red boxes highlight pollen grains with morphological abnormalities. The *hb8* mutant was *SlHB8-cr3*; NT: normal temperature; LT: low temperature; MI: microspore mother cell stage; Tds: tetrad stage; MUM: middle uninucleate microspore stage; BM: binucleate microspore stage; MP: mature pollen stage; MSP: microspore; T: tapetum; Td: tetrad. APG: abnormal pollen grain; PG: pollen grain.

To more accurately distinguish the stages of pollen development, we classified flower buds into eight flowering stages based on their morphology and size, ranging from 2 to 8 mm. We confirmed the accuracy of these stages by using DAPI staining to examine the microspore size by microscopy. The results showed that at room temperature, there were no noticeable differences in pollen morphology between wild-type and *hb8* mutant plants throughout various developmental stages. However, under cold stress conditions, both WT and *hb8* mutants formed normal tetrads during the microspore stage, but abnormalities appeared in WT pollen after this stage; pollen grains with no visible nuclei and shrunken appearances were noted (Figure 4a). In contrast, the *hb8* mutant pollen maintained a normal appearance (Figure 4a).

To examine the morphology of the pollen and tapetum across various developmental stages at both room and cold temperatures, we collected samples based on bud size for paraffin sectioning. The results showed that under room temperature conditions, there were no noticeable differences in anther morphology between the wild type and *hb8* mutants throughout their development. However, under cold treatment, the WT anthers did not exhibit significant changes during the microspore mother cell stage (MI); but at the tetrad stage (Tds), the WT tapetum became swollen with abnormal tetrads forming, unlike in *hb8* mutants which appeared normal; by the middle uninucleate microspore stage (MUM), the WT microspores began to wither and shrink while *hb8* maintained many normally developing microspores; finally, at the pollen maturation stage (MP), almost no normal pollen grains remained in WT plants, whereas *hb8* mutants had a high amount of full granules with typical morphology and viable pollen (Figure 4b). The findings from these sections indicate that the loss of function of SlHB8 does not impact pollen development under normal conditions. However, cold stress causes developmental defects in the tapetal cells and microspores of wild-type plants. In contrast, *hb8* mutants lacking SlHB8 exhibit increased resistance to cold during anther development, maintaining normal tapetal degradation and continuous microspore development under cold conditions.

**Figure 5:**
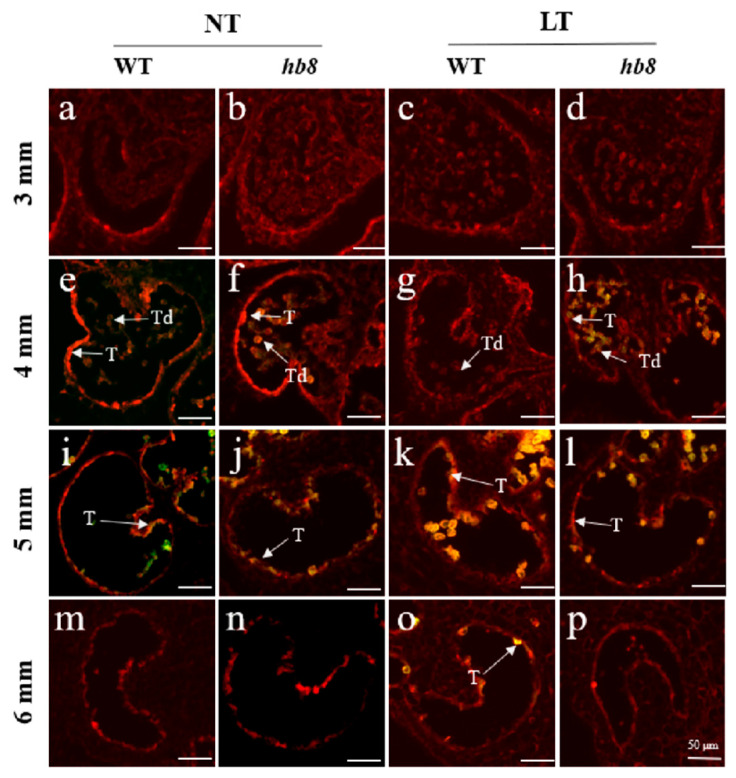
Fluorescence microscopy of DNA fragmentation detected by TUNEL assays of anthers from the wild type and *slhb8* mutant under both normal and cold stress. (**a**,**e**,**i**,**m**) represent wild type anthers of 3 mm, 4 mm, 5 mm, and 6 mm length at normal temperature; (**b**,**f**,**j**,**n**) represent *slhb8* anthers of 3 mm, 4 mm, 5 mm, and 6 mm length at normal temperature; (**c**,**g**,**k**,**o**) represent wild type anthers of 3 mm, 4 mm, 5 mm, and 6 mm length under low temperature; (**d**,**h**,**l**,**p**) represent *slhb8* anthers of 3 mm, 4 mm, 5 mm, and 6 mm length under low temperature; The *hb8* mutant was *SlHB8 -cr3*; 3 mm: flower bud of 3 mm length; 4 mm: flower bud of 4 mm length; 5 mm: flower bud of 5 mm length; 6 mm: flower bud of 6 mm length; T: tapetum; Td: tetrad; Scale bars = 50 μm.

The programmed cell death of tapetal layer cells provides lipids, proteins, and precursor substances for microspore development; premature or delayed degradation of the tapetum leads to pollen abortion. Studies indicate that under cold conditions, delayed tapetal degradation in anthers contributes to pollen sterility due to the failure of providing necessary nutrients for microspore development. To determine whether the cold resistance of *hb8* mutant pollen is related to tapetal degradation, we compared the degradation of the tapetum in both the wild type (WT) and *hb8* mutants under normal and cold temperatures using TUNEL assays. Under normal conditions, tapetal layer degradation signals appeared at the tetrad stage (4 mm) for both WT and *hb8* mutants, intensified in the early uninucleate stage (5 mm), and disappeared by the late uninucleate stage (6 mm) (Figure 5). Under cold treatment, WT showed no tapetal degradation signal at the tetrad stage (4 mm), with strong degradation signals emerging at the early uninucleate stage (5 mm) and persisting into the late uninucleate stage (6 mm). In the *hb8* mutant, the tapetal degradation signals were similar to those under normal conditions, without any detectable delay (Figure 5). This suggests that SlHB8 is related to cold-induced delay in tapetal layer degradation.

### 2.4. Transcriptome Analysis of DEGs in Anthers of the Wild Type and hb8 under Cold Treatment

To further characterize the role of SlHB8 in regulating cold tolerance in anthers, we analyzed and compared the transcriptome profiles of WT and *hb8* anthers at the tetrad stage under both normal and low-temperature conditions. Using a threshold of a fold change ≥ 2 and an FDR ≤ 0.05 to identify differentially expressed genes, we found that there were a total of 1056 differentially expressed genes responded to low temperature in WT after cold treatment, with 471 genes being upregulated and 585 being downregulated (Figure S1a; Table S1). In the *hb8* mutant, a total of 1692 genes were differentially expressed before and after cold treatment, with 636 genes upregulated and 1056 genes downregulated (Figure S1a; Table S1). At normal temperature, in the tetrad stage anther tissue of WT and *hb8*, there were 730 differentially expressed genes, with 599 genes showing upregulation and 131 genes downregulated (Figure S1a). After 10 days of cold treatment, the number of differentially expressed genes reduced to 380, with 210 genes upregulated and 170 genes downregulated (Figure S1a).

**Figure 6:**
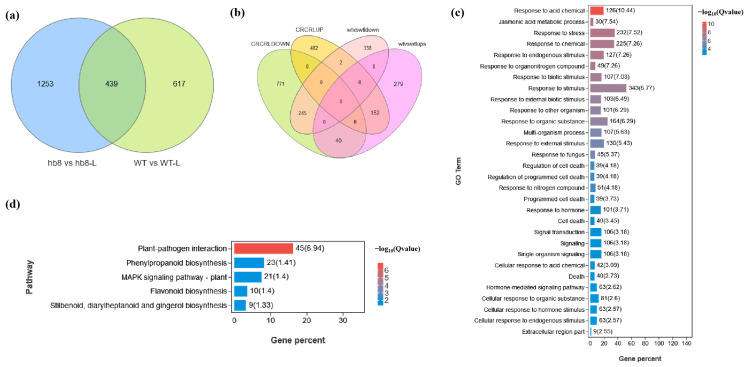
Identification of differentially expressed genes (DEGs) responsible for the cold resistance in *slhb8* anthers. (**a**) Venn diagram analysis of genes differentially expressed in WT and *slhb8* before and after cold treatment; (**b**) Venn diagram analysis of genes with opposite expression trends in WT and *slhb8* before and after cold treatment. (**c**) GO analysis of specifically differentially expressed genes in *hb8* (1253 + 42 = 1295); (**d**). KEGG pathway analysis of the specifically differentially expressed genes (1295) in *hb8*. The *hb8* mutant was *SlHB8-cr3*.

The knockout of SlHB8 has been found to enhance the cold resistance of tomato pollen. Genes that are specifically differentially expressed in *hb8* before and after cold treatment may be associated with cold resistance. A Venn diagram analysis identified 1253 genes with unique differential expression patterns in *hb8* (Figure 6a). Additionally, there are 42 genes that both WT and *hb8* share; however, these genes show opposite trends in response to cold treatment in WT and *hb8* (Figure 6b), resulting in a total of 1295 genes that respond specifically to low temperatures in *hb8* (Table S1). GO and KEGG pathway analyses of these 1295 DEGs indicate significant enrichment for functions related to stress response and metabolic pathways: the GO terms include those associated with stress response (GO:0006950), jasmonic acid metabolism (GO:0009694), the regulation of cell death (GO:0010941), programmed cell death processes (GO:0043067, GO:0012501), the cellular responses to acidic chemicals (GO:0071229), and hormone-mediated signaling pathways (GO:0009755) (Figure 6c); while enriched KEGG pathways cover phenylpropanoid biosynthesis (ko00940), plant–pathogen interactions (ko04626), flavonoid biosynthesis (ko00941), stilbenoid/diarylheptanoid/gingerol biosynthesis (ko00945), and MAPK signaling pathway (ko04016), among others (Figure 6d).

Among the 1295 unique genes identified as differentially expressed, the activity of 375 DEGs is dependent on the SlHB8, suggesting that SlHB8 may enhance cold resistance in *hb8* through the regulation of these genes (Figure S1b). Gene ontology (GO) and Kyoto Encyclopedia of Genes and Genomes (KEGG) analyses showed that these 375 genes are significantly enriched in GO terms such as stress response (GO:0006950), jasmonic acid metabolism (GO:0009694), cell death regulation (GO:0010941, GO:0043067, GO:0012501, GO:0008219), and the cellular response to acidic chemicals (GO:0071229) (Figure S1c). The KEGG pathway enrichment includes plant–pathogen interaction (ko04626), taurine and hypotaurine metabolism (ko00430), flavonoid biosynthesis (ko00941), and the MAPK signaling pathway (ko04016) as notably significantly enriched pathways (Figure S1d).

### 2.5. Knocking Out SlHB8 Blocks Cold-Induced Activation of DYT1-TDF1-AMS-MYB80

**Figure 7:**
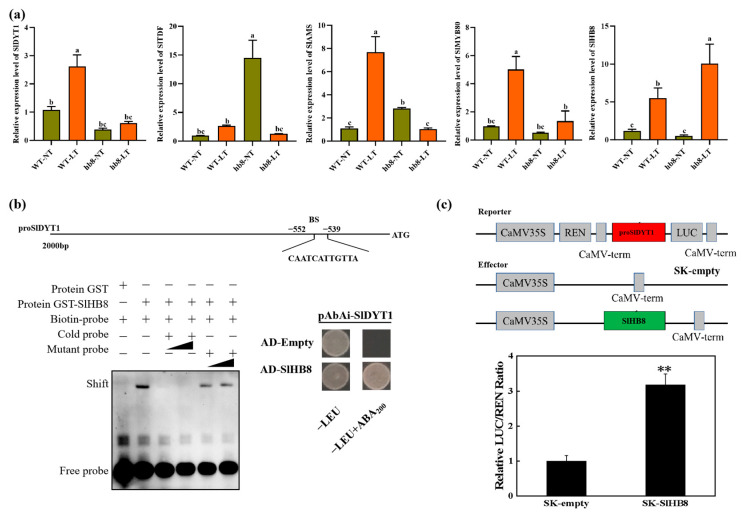
The regulation of SlHB8 in the tapetum PCD process. (**a**) The expression pattern of tapetum PCD regulators in anthers of WT and *slhb8* mutant under NT and LT conditions. The *hb8* mutant was *SlHB8-cr3.* Each error bar represents the mean ± SE, *n* = three biological replicates. Data are expressed as the mean ± standard deviation. Significant differences in mean values are indicated by the letters a–c: Tukey HSD, *p* < 0.05. (**b**) Validation of SlHB8 binding with the promoter of *SlDYT1* by using a yeast one-hybrid assay and EMSA assay. AD-empty indicates the control yeast strain transformed with the empty pGADT7 vector without SlHB8. EMSA showing the binding of SlHB8 to the promoters of *SlDYT1* via the BS. The probes containing the BS were labeled with biotin. Competition for SlHB8 binding was performed with 5× and 20× cold probes containing the BS or mutated (CAAAAAAAAAA) controls. −: absence; +: presence. (**c**) Validation of SlHB8 activation on the SlDYT1 promoter by using a dual-luciferase assay. The empty effector was used as a control (set as 1). The ratio is presented as the mean ± SE (*n* = 3). Significant differences in mean values are indicated by an asterisk: ** *p*-value < 0.01; Student’s *t*-test.

The degradation of the tapetum layer was affected by the cold treatment, and the genes deferentially expressed specifically in *hb8* before and after cold treatment were significantly enriched in the pathway of programmed cell death. Consequently, we analyzed the expression patterns of the key regulators in the tapetal degradation pathway, DYT1-TDF1-AMS-MYB80, and the results showed that under cold treatment, the expression level of *DYT1*-*TDF1*-*AMS*-*MYB80* increased in the wild type, while they failed to increase in *hb8*, indicating that SlHB8 involvement is essential for regulating *DYT1*-*TDF1*-*AMS*-*MYB80* during the response to low temperatures (Figure 7a).

Upon analyzing the promoter sequence of *SlDYT1*, we found a binding site for SlHB8 (Figure 7b). Then, we performed Y1H and EMSA to confirm the binding of SlHB8 with the *SlDYT1* promoter. Meanwhile, we also carried out dual luciferase assays to confirm the regulation of SlHB8 on the *SlDYT1* promoter. The results showed that SlHB8 could bind the promoter of *SlDYT1* and activate the expression of *SlDYT1* (Figure 7b,c), indicating that SlHB8 may serve as an upstream regulator of SlDYT1 and influence tapetum layer degradation by modulating its expression (Figure S2 and Figure 7).

### 2.6. The SlHB8 Interact with SlTDF1-1 and SlMYB80

**Figure 8:**
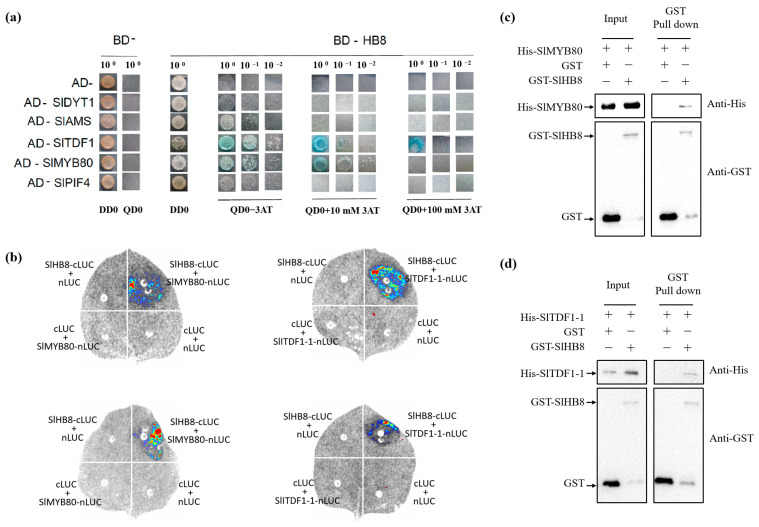
The interaction between SlHB8 and the tapetum PCD regulators. (**a**). Yeast two-hybrid (Y2H) interaction between SlHB8 and proteins involved in tapetum development and degradation. Yeast growth of co-transformed BD-SlHB8 and AD-SlDYT1/SlAMS/SlTDF1/SlPIF4/SlMYB80. The yeast clones grown on selected medium lacking Trp, Leu, His, and Ade (TLHA) were inoculated again on a TLHA plate. The plate was added with 3AT accordingly. After 3–4 (**d**), the growth of the yeast strains confirmed a positive interaction, as shown. (**b**). Luciferase complementation assay in tobacco leaf epidermal cells showing the interaction between SlHB8 and SlTDF1, SlMYB80 in living cells. SlTDF1 and SlMYB80 were fused with the N-terminus of LUC (NLuc), SlHB8 was fused with the C-terminus of LUC (CLuc), as indicated, and co-transfected into N. *benthamiana* leaves by A. *tumefaciens* infiltration. The luminescence images were captured using a CCD imaging system. The images at the top and the bottom respectively illustrate the interactions between SlHB8 and SlMYB80/SlTDF1 under normal and low temperature conditions. (**c**,**d**). Pull-down assay for detecting the interaction between SlHB8 and SlMYB80, SlHB8 and SlTDF1.

We co-transformed various pGADT7 recombinant plasmids (AD-SlDYT1, AD-SlTDF1, AD-SlPIF4, AD-SlAMS, AD-SlMYB80) with the BD-SlHB8 plasmid into the AH109 yeast strain to test for protein interactions. The BD-SlHB8 showed minor self-activation which was completely suppressed at a 10 mM concentration of 3AT. Notably, yeast strains harboring both AD-SlTDF1and BD-SlHB8 or AD-SlMYB80 and BD-SlHB8 grew on quadruple dropout media containing 10 mM 3AT and exhibited blue colonies during X-alpha-gal screening (Figure 8a). In contrast, the negative control only grew on double dropout media but not on quadruple dropout plates. This suggests that within the yeast system, there are specific interactions between SlHB8 and both SlTDF1 and SlMYB80 proteins; however, no interaction was detected between SlHB8 and either SlDYT1, SlPIF4, or SlAMS (Figure 8a). These findings were further confirmed by a luciferase complementation assay and pull-down that validated the interactions between SlHB8 with SlMYB80 as well as SlTDF1 (Figure 8b–d).

## 3. Discussion

### 3.1. SlHB8 Exhibited a Critical Influence on the Cold Resistance of Tomato Pollen, Primarily by Regulating the Delayed Degradation of the Tapetum under Low Temperature

Tomato originates from tropical or subtropical regions and is highly sensitive to cold stress [36]. Cold temperatures often result in lower tomato yields and quality, as cold disrupts the plant’s reproductive process, especially affecting male fertility stages [4]. However, our understanding of the potential regulatory mechanisms is very limited; thus, investigating the response mechanisms of male development in tomatoes to cold stress is crucial for the genetic improvement of this crop.

SlHB15A is a member of the HD-zip III family of transcription factors. A loss-of-function mutation in *SlHB15A* enhances the tomato fruit-set rate under low temperatures. The HD-zip III family is negatively regulated by *miR166*, and SlHB15A regulates parthenocarpic fruit formation at low temperatures through *miR166* mediation [32]. Our previous research indicated that *SlHB8*, a member of this family, affects several aspects of tomato plant anatomy. A lack of SlHB8 activity led to thicker stems and shorter plants [33], while its overexpression resulted in rolling leaves, thinner stems, and pollen abortion [33,34,35]. Specifically regarding pollen development, increased levels of *SlHB8* triggered premature tapetal breakdown and consequently pollen sterility [33]. Given its negative regulation by *miR166*, we hypothesized that *SlHB8* might also influence pollen development at low temperatures. In our study, tomatoes exposed to low-temperature conditions (15 °C/10 °C) exhibited reduced pollen viability, pollen tubes germination rates, and fruit-set rates (Figure 2). In wild-type plants subjected to this condition, there was a significant decrease in these traits as well as in fruit size; this led to smaller fruits of lower quality and the increased production of seedless fruits. By contrast, *hb8* mutants maintained higher levels of pollen viability and pollen tube germination rates than the wild type; they also had better fruit-set rates along with larger fruits containing more seeds (Figure 2). These findings indicated that SlHB8 acts as a negative regulator for cold tolerance in tomato anthers; knocking out *SlHB8* can improve the pollen viability of tomatoes under low temperatures, thereby increasing the fruit-set rate, yield, and quality.

Cold stress can cause pollen sterility due to dysfunctional tapetal behavior during the microsporogenesis phase, as documented by various researchers [22,35]. Either an early or delayed degradation of the tapetum significantly disrupts microspore growth and maturation. This delay, along with issues such as cell enlargement and vacuolation in the tapetum, leads to pollen infertility [37,38]. In tomatoes, low-temperature exposure delays programmed cell death (PCD) signals in the wild-type tapetum, hindering microspore development and causing pollen degradation [31]. In our study, DAPI staining and paraffin sectioning were performed to analyze the morphology of microspores and tapetal cells in the anthers of WT and *hb8* mutant under both normal and low-temperature conditions. At normal conditions, there were no significant differences in the pollen microspores and anthers of WT and *hb8* through all the tested developmental stages. Under cold stress, however, abnormalities became apparent in the WT tapetum at the tetrad stage, with noticeable cell enlargement, and remnants of the tapetum were still detectable at the binucleate stage, while the *hb8* mutant did not exhibit significant abnormalities (Figure 4). TUNEL assays indicated that the programmed cell death signals in both WT and *hb8* tapetal cells began at the 4 mm stage, continued through 5 mm, but decreased by 6 mm under normal conditions. In contrast, low temperatures induced a delayed degradation signal in the WT tapetum, with positive signals only emerging at the 5 mm stage and persisting at the 6 mm stage; this delay, however, was not seen in *hb8* mutants (Figure 5). These results are consistent with previous research suggesting that colder temperatures can postpone tapetal degradation, leading to pollen sterility. Additionally, transcriptomic data from the tetrad stage anthers of both the wild type and *hb8* under cold treatment revealed a significant enrichment of differentially expressed genes (DEGs) specific to the *hb8* in pathways modulating cell programmed death (Figure 6c), suggesting that SlHB8 may play a role in regulating the tapetal cell programmed death process under cold conditions. Combining the results of cytological analysis, it is proved that the *hb8* mutant conferred cold tolerance to tomato pollen by preventing the delayed degradation of tapetal cells in cold conditions.

### 3.2. SlHB8 Enhanced Cold Tolerance in Anthers through Interaction with the Tapetum Regulatory Module

In *Arabidopsis*, researchers have identified a series of transcription factors essential for tapetum development and degradation. The DYT1-TDF1-AMS-MYB80 genetic pathway has been shown to control programmed cell death (PCD) of the tapetum in Arabidopsis and is thought to be functionally conserved across rice, maize, and tomato species [27,28,29,30]. DYT1, an upstream bHLH transcription factor in the tapetal regulatory network, enhances downstream target gene expression when activated. AMS and MYB80 are key transcription factors following TDF1 that directly regulate genes involved in callose dissolution, sporopollenin production, lipid transfer, and pollen wall formation during pollen maturation. However, there is a significant gap in our understanding of the molecular mechanisms that confer low-temperature tolerance during tapetal cell degradation. In tomato, the tapetal PCD regulatory module *DYT1*-*TDF1*-*AMS*-*MYB80* responds to low temperatures with increased expression following cold stress [31]. The bHLH transcription factor SlPIF4 directly interacts with this module and negatively affects anther cold resistance under such stress. Research has shown that low temperatures prompt the formation of a SlPIF4–SlDYT1 complex, which in turn enhances the activation of *SlTDF1*’s transcription. This leads to delayed tapetum degradation and subsequent pollen sterility. In contrast, *slpif4* mutants show suppressed cold-induced activation of *SlTDF1*, resulting in improved pollen cold resistance [31].

In our study, under cold treatment, the expression of *DYT1*-*TDF1*-*AMS*-*MYB80* increased in the wild type but failed to increase in the *hb8* mutant (Figure 7), suggesting that the response of *DYT1*-*TDF1*-*AMS*-*MYB80* to low temperatures is mediated by SlHB8. Additionally, SlHB8 is a transcriptional activator and the promoter of *SlDYT1* and *SlMYB80* contains binding sites of SlHB8; yeast one-hybrid, EMSA and dual-luciferase assays have demonstrated that SlHB8 is capable of directly binding to and activating the promoter of *SlDYT1* and *SlMYB80* [33]. Therefore, after exposure to cold, wild-type plants exhibit an increase in *SlDYT1* and *SlMYB80* expression due to activated *SlHB8*; this upregulation does not occur in *hb8* mutants, resulting in inhibited signals for delayed tapetal degradation under cold stress (Figure S3). The interactions between the tapetal PCD regulatory factors DYT1-TDF1-AMS-MYB80 and the SlHB8 protein were investigated using yeast two-hybrid experiments, pull-down assays, and dual-luciferase complementation assays. These studies confirmed that SlTDF1 and SlMYB80 interact with SlHB8 individually (Figure 8b–d), suggesting the possibility that SlHB8 might regulate tapetal PCD at the protein level.

## 4. Materials and Methods

### 4.1. Plant Material and Cold Treatment

In this study, wild-type Micro-tom and *slhb8* mutant plants obtained through CRISPR/Cas9 gene editing technology with Micro-Tom as the background were utilized as experimental materials. These *slhb8* mutants were first identified in Wu et al.’s 2022 paper [33]. We monitored flower buds on both the wild type and *SlHB8-cr1*, *SlHB8-cr2*, and *SlHB8-cr3* mutants until they reached the tetrad stage (3–4 mm) in their microspore development. To keep track, we marked these buds using a straw on the pedicel. We marked at least twenty plants of each genotype. Half of these selected plants underwent seven days of low-temperature treatment at 15 °C/10 °C in a growth chamber, while the others were left as controls at standard artificial greenhouse temperatures (25 °C/20 °C) with sixteen light and eight dark hours. The plants grew in 10 cm × 10 cm pots filled with an imported peat moss and perlite mixture at a 2:1 ratio. For watering, we alternated between mixed tap water and a Hyponex No. 2 nutrient solution with tap water in a 1:1000 ratio, every 2–4 days.

### 4.2. Pollen Viability and Pollen Germination Assay

Anthers at the anthesis stage were gathered from plants exposed to both normal and low-temperature conditions to evaluate pollen viability and germination rates. The methods detailed in Wu et al., 2022, were used to measure pollen viability and germination [33].

### 4.3. Fruit Set and Fruit Morphology Analysis

Once the fruits from the buds, marked and set under normal and low-temperature conditions at the tetrad stage, ripened, we calculated the fruit-set rates for each genotype. The fruit-set rate refers to the percentage of marked flowers that turn into fruit. After determining the fruit-set rate, we harvested the marked fruits and recorded their size, weight, and seed number for WT, *SlHB8-cr1*, *SlHB8-cr2*, and *SlHB8-cr3* under both temperature treatments.

### 4.4. DAPI Staining

Stages of microsporogenesis were determined by sampling various flower sizes, ranging from 2 mm to 8 mm, as well as on the day of flowering, after stopping the low-temperature procedure. Next, a glass slide was prepared with 60 µL of DAPI solution. The anthers were cut horizontally and steeped in DAPI solution. Using forceps, we gently pressed to free the pollen. We covered the sample and left it to stain in the dark for five minutes [39]. Finally, we examined the morphology of the microspores under a fluorescence microscope and documented the results with photographs.

### 4.5. Cytological Characterization of Anthers

Anthers at various development stages (MI, microspore mother cell stage; Tds, tetrad stage; MUM, middle uninucleate microspore stage; BM, binucleate microspore stage; MP, mature pollen stage) were gathered from plants during the flowering period under both normal and cold conditions. The samples were then left to fix at room temperature for 24 to 36 h. Following paraffin embedding and sectioning, anther cells were analyzed using a Leica microscope. The DeadEnd™ Fluorometric TUNEL System (Promega, Madison, WI, USA) was utilized for conducting a terminal deoxyribonucleotidyl transferase-mediated biotin-16-dUTP nick-end labeling (TUNEL) test, according to the system’s guidebook. Images of the sections were captured with a Leica TCS SP5 fluorescence confocal scanning microscope. Finally, emission wavelengths of 488 nm/505–545 nm and 561 nm/575–650 nm were used to detect TUNEL and propidium iodide signals.

### 4.6. In Situ Hybridization Assay

We analyzed wild-type floral buds at the Tds stage, about 4 mm in size, under normal and low temperatures using in situ hybridization. Both the sampling and fixation methods were the same as those used for the paraffin-sectioned specimens. We used a special in situ hybridization fixative from Servicebio for fixation. Following Liu et al.’s 2022 guidelines [34,35], we used both positive and negative probes during hybridization. An optical microscope was used for observation and photography.

### 4.7. RNA-seq and qRT-PCR Analysis

We collected flower buds at the tetrad stage from plants under both normal and cold conditions. These samples were then frozen using liquid nitrogen in preparation for RNA extraction and transcriptome sequencing. This experiment will be conducted by Gene Denovo Biotechnology Co., Ltd. (Guangzhou, China). Sequencing libraries were generated using the NEBNext Ultra^TM^ RNA Library Prep Kit for Illumina (NEB, Ipswich, MA, USA) following the manufacturer’s recommendations, and index codes were added to attribute sequences to each sample. The library preparations were sequenced on an Illumina platform HiSeqTM 2500 and paired-end reads were generated. Aiming to obtain high-quality clean reads, reads generated from the sequencing machines were further filtered by fastp (version 0.18.0). The reads containing adapters, containing more than 10% of unknown nucleotides and containing more than 50% of low-quality bases were removed. The raw and clean reads obtained are listed in Table S2. The filtered high-quality reads were assembled and mapped to the SL4.0 genome (https://solgenomics.net/organism/Solanum_lycopersicum/genome/, accessed on 14 November 2024), using HISAT2 (version 2.0.4) and StringTie (version v1.3.1; the parameters were the default). To calculate the expression levels of the identified genes, we used the fragments per kilobase of transcript per million mapped reads (FPKM) by RSEM software (version 0.6). The differentially expressed genes (DEGs) analyses were performed by DESeq2 software (http://www.bioconductor.org/packages/release/bioc/html/DESeq2.html, accessed on 25 August 2024) between two different groups. DEGs were defined by a log2 (fold change) ≥1 and a false discovery rate (FDR) ≤ 0.05. GO enrichment analysis identifies GO terms that are significantly enriched in DEGs compared to the genome background and filters the DEGs that correspond to biological functions. Firstly, all DEGs were mapped to GO terms in the Gene Ontology database (http://www.geneontology.org/), accessed on 14 November 2024, gene numbers were calculated for every term, and significantly enriched GO terms in DEGs compared to the genome background were defined by hypergeometric test. The calculated *p*-value underwent FDR correction, taking FDR ≤ 0.05 as a threshold. GO terms meeting this condition were defined as significantly enriched GO terms in DEGs. Similarly, KEGG pathway enrichment analysis identified significantly enriched metabolic pathways or signal transduction pathways in DEGs compared with the whole genome background. The calculated *p*-value was gone through FDR correction, taking FDR ≤ 0.05 as a threshold. Pathways meeting this condition were defined as significantly enriched pathways in DEGs. The analysis and mapping of transcriptome data were carried out on the OmicShare Tools online platform developed by Gene Denovo (www.omicshare.com/tools), accessed on 14 November 2024. We generated heatmaps using TBtools, following the method outlined in the 2020 Chen et al. manual [40]. We used qRT-PCR to verify the reliability of the RNA-seq. The SYBR PrimeScript^TM^ RT PCR Kit II (Takara Bio, Kusatsu, Japan) and sequence-specific primers were utilized, with ubiquitin (UBI; ID: Solyc01g056940) serving as the reference gene. The relative expression levels of the genes under study were determined using the 2^−ΔΔCT^ method, taking into account three biological replications.

### 4.8. Yeast One-Hybrid Assay (Y1H)

We used the pGADT7-Rec vector, containing the full-length CDS of SlHB8, as a prey vector and the pAbAi vector with SlHB8 binding elements from SlTDF1 promoter as a bait vector. After transforming Y1H Gold yeast strains with linearized pAbAi constructs, we determined the minimal inhibitory concentration of Aureobasidin A. The binding activity of SlHB8 on the elements was checked by transforming the prey vector into the bait yeast strains, and then culturing it on SD medium without Leu (SD/-Leu). This culture was prepared either with or without Aureobasidin A at a pre-selected concentration, and it was kept at 30 °C for 2–3 days.

### 4.9. Dual-Luciferase Transient Expression (DLR) Assay

To evaluate the regulatory function of SlHB8 on the promoter SlDYT1 with SlHB8 binding elements, we used the pGreenII 62-SK vector containing the SlHB8 CDS as the effector vector, and the pGreenII 0800-LUC vector with target promoters as the reporter vector. These diverse plasmids were injected into *Nicotiana benthamiana* leaves through *A. tumefaciens* mediation. We then measured the luciferase and Renilla activities using the Dual-Luciferase Assay Kit (Promega), following the procedures outlined in its manual.

### 4.10. Yeast Two-Hybrid (Y2H) System

The full SlHB8 CDS was cloned into the pGBD vector (BD-SlHB8 by Clontech). Similarly, the full CDSs of tapetal PCD-associated genes (SlDYT1, SlTDF1, SlAMS, SlMYB80, SlPIF4) were cloned into Clontech’s pGAD (AD-genes) vectors. The procedure followed was detailed by Guan et al. (2018) [41]. The genes are identified by the IDs SlHB8 (Solyc08g066500), SlPIF4 (Solyc07g043580), SlDYT1 (Solyc02g079810), SlTDF1 (Solyc03g059200), SlAMS (Solyc08g062780), and SlMYB80 (Solyc10 g005760).

### 4.11. Luciferase Complementation (LCI) Assay

The luciferase complementation assay was carried out according to the methodology established by Zhou et al. (2018) [42,43]. The full-length coding sequences of SlHB8, SlTDF1, and SlMYB80, excluding stop codons, were fused with NLuc and CLuc, respectively, then inserted into the pCAMBIA1300-NLuc and pCAMBIA1300-CLuc vectors, correspondingly.

### 4.12. Electrophoretic Mobility Shift Assay (EMSA)

*SlHB8* was ligated into a pGEX4T1 vector and named SlHB8-GST. SlHB8-GST was transformed into BL21 (DE3) *E. coli* for protein induction purification at 16 °C for 16 h. A DNA double-stranded probe (Wild type: GTCTATATTCAATCATTGTTATGCTATATGC; Mutant: GTCTATATTAAAAAAAAAAAAAGCTATATGC) containing the SlHB8 transcription factor binding element was designed on the promoter of *SlDTY1* and labeled with biotin, followed by EMSA binding reaction using a thermo kit, and then imaged and analyzed after electrophoresis, membrane transfer, and color development.

### 4.13. Pull-Down Assay

*SlMYB80* and *SlTDF1-1* were ligated into a pET32a vector and named SlMYB80-his and SlTDF1-1-his, respectively. SlMYB80-his and SlTDF1-1-his were transformed into BL21 (DE3) *E. coli* at 16 °C for 16 h for protein induction purification. SlHB8-GST and GST-null were incubated with GST-tagged protein purification magnetic beads, and then SlMYB80-his and SlTDF1-1-his proteins were added for incubation. This was followed by electrophoresis, closure, the incubation of antibodies, and development and photographing.

## Data Availability

All data generated or analyzed during this study are included in this published article and its Supplementary Information Files. The original RNA-seq data presented in the study are publicly available. These data can be found here: China National Center for bioinformation (https://bigd.big.ac.cn/gsa/), accessed on 22 August 2024, accession number CRA018478.

## Supplementary Materials

The following supporting information can be downloaded at https://www.mdpi.com/article/10.3390/ijms25179336/s1.
