# Supplementary material for: SlHB8 Is a Novel Factor in Enhancing Cold Resistance in Tomato Anthers by Modulating Tapetal Cell Death
Source: Int J Mol Sci. 2024 Aug 28;25(17):9336. doi: 10.3390/ijms25179336 (PMC11395002; doi:10.3390/ijms25179336)
Supplement: Supplementary file 1 [file ijms-25-09336-s001.zip › ijms-3121030-supplementary.pdf]

Supplementary Information

Article title: SlHB8 is a Novel Factor in Enhancing Cold Resistance in Tomato Anthers by Modulating Tapetal Cell Death

Authors: Hongling Guan, Canye Yu, Zaohai Zeng, Huimin Hu, Yuxiang Lin, Caiyu Wu, Yiwen Yao, Rui Xia, Zhengguo Li, Chongjian Ma, Riyuan Chen, Baowen Huang*, Yanwei Hao*

The following Supporting Information is available for this article:

Figure S1–S4

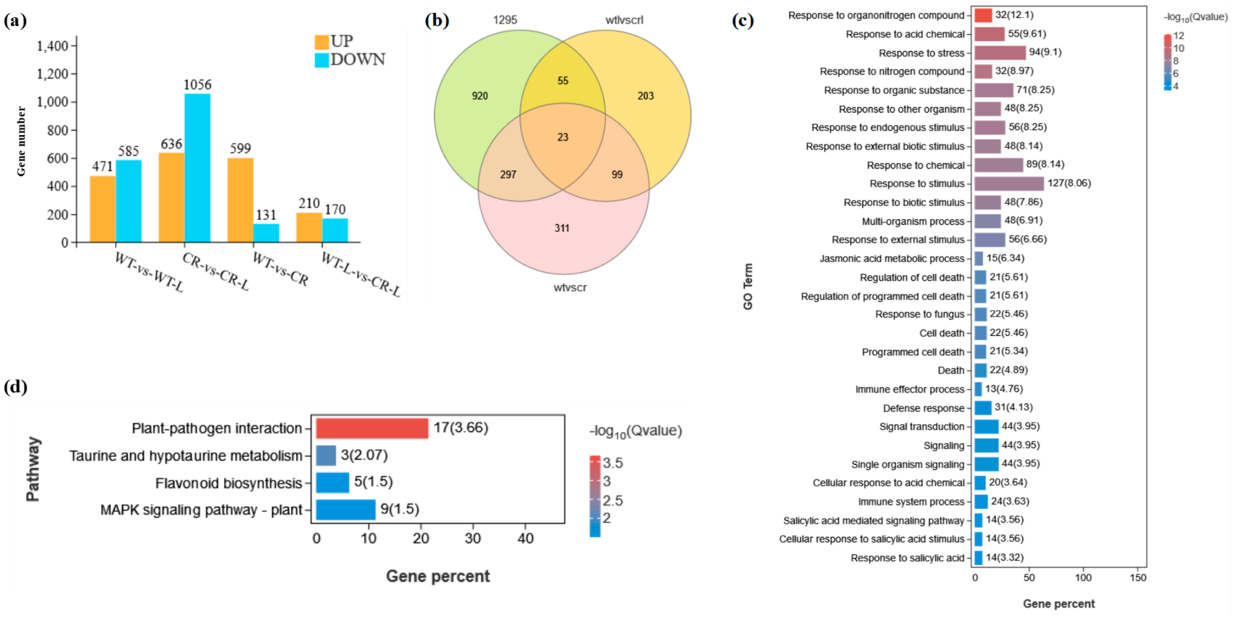

Figure S1. Identification of differentially expressed genes (DEGs) in wild-type and *slhb8* anthers under normal and cold treatment by RNA-seq.

a DEGs among wild-type and *hb8* anthers at the tetrad stage under normal and cold conditions. **b** Venn diagram analysis of genes that specifically differentially expressed in *hb8* (1253+42=1295) were simultaneously differentially expressed between wild type and *hb8* mutant under normal and cold treatment (297+32+55=375). **c** GO analysis of 375 DEGs; d. KEGG pathway analysis of the 375 DEGs. The *hb8* mutant was *SlHB8-cr3*.

GATCATTTGAAATTAGAACACTTCGTAAAACACACTATTTAGGTGATCTCTTCTTATTTTTTATAACAGTGATATCTAGACTAATTAATACACACATTAATTAATTGTACCAAGGGCTTGATC
ATCTCAAAATTTTGTGCACACCAACAATCCAAATACCTTTTCCCTTCTAAAACTACCTTGGGGTGTAACAATAAAATCCTTGCATTCACATACTTGGCATTTTCAAGTGAGAATGTAGATTAT
GCAAAATTTTGCAAACATTATTAATCTCTTAATACATAAATGCAAGGATACCAACATCTAATTTATACAAGTCCAGCACCAGCATCCTTTTAAAATGTTTAGATCCTCCAACTTGATTGAATG
TCAACAGTCAAAATGTAACATACTTTAACCTAAGTGCAACAAGCTAAATTTTTTGTATAGAGTATATATCCGACCATTCCTTAAACCCTTCATCACTTCACCTAGCTCAAGTAAACAATCAAA
TCACGTAGCTGAAAGAACTAGTTATGCTAAGGCAAGGAAAGTTCATTCCGAAAGCTCTTATAAGACTGAGCCAACTTAACCAGTTCTCTCGCAGTTTCAATCTTAGAATGCTCAACCAAACAA
TTGACAACCCTCTGCATTATTGTATTATGAATAGCTTTACGTGACTTCATTGCTTCCTTGCACAAATCCAGAGCAAAATCGACTTTTTTGGCGCTACAAGCAAAATTAACAAGTATGGTGAAT
GTTGAAACATCCGGAAGACATCCGTTTTGCGTCATCTTCTCATACCAAATGTTGACCTCCTCCAGATTCCCCTTATTAACATACATTTTTATCATAGCACTGTAACTGAAATTTATCCGGTTT
GAAACCCTTGTTAACAGTCTCATCGAAGAACTGAATCGCCTTTGAAACCTCGTTGGCTTTAACCAAACTAATTAATTTAATATTGTGACTGCAAAGATCGGGGATAATATTTCTCTTTTCCAT
CATAGCCCACAAATTTTCAGCTTCAGAAAACCTTTTTCTTTCATAAAATGCGGTTAATGACGTGTTACAAGTGACAATATTCGGCTTAATCCCATGATTTTCAATCTCATCCATCAAATACAC
AGCAGAATCCAACAAACCAGCCCTGCACAGTGCCTTAATCGCAGTGGCATACGACACCAGATCAGGCTGAATTGACAATTTCGGGGGCAATTCGCGAAACAAGCCGCCAATTTCATCATATTT
CTCTGATTTAAGATATGCTTCCAGTAGGGCATTGAAGGAAAAGACAGTGCGCGGACAATTTAGGTGAGGCATTTCGTCAAACAGCTTACGTGCACGATGATCCATTTTCGCTCTGCCATAGAG
AAGAATAAGACGGGCAACAAAGGCTTCGTTACGAATATCAGGATACTTTTTTTTGATGTTCAATGATATCTATAATGCCGGATTTGTGTTTGGCTTCGGTAAGTTGGTGAATGGCGATTTCAT
AATGGAAGCGCGATTCAGTCGGAATTTGGGGCATTCCGATGATTTTTTGAAAGAGTCTACCATTGCTTGGGCTCGATCGGTGGGTCTTGAACAAGTAATGATCTGATTTATTGCTCTTTCATT
TCCGGCGAAGATGCCTTTCACACGGCGGAGAGATGAAGAAGTAGACATTTGATGCTTATTTTGATTTTTGGCGCTGAGCAAGACGTTAGAAAGAAAGAAAAAATTCAATTATACCCCTAACGT
TTATTATTTGGTTTATATATGGCATTTGTTAACTTACCGCATAATAGCTGAAAGAATCTGATTATTTTGGTACAATAAATTACAACAATAAAACAAAGAGCCTTATTCAACGTTGTTATGTTA
CGAAGGACTTATGTGATACTTCCTGAAGAGTAAAAGCTCCTATGGCCTTTTCCCATACCAGCATTATAAGTTGTTGCTTCGTATTTTTGTATTTGTATTTAATTTCTCTTTTTTATAAATAAA
TGATAAAATTACATTCACATCATCTACACTAGATATATTGTTGTATCATATTTTGGTTTTTGAGTGGTTAAAGTACAAAATACATACATGGAATACCATTAACTCTTTCTAGTGATTTACAAA
ATGAAGTTGTACTTTAAAAGAAATTATTCATGTCAGGTGTAAAAAAGAAAAAGTGAAAGGAGAATAAAATTTCTACTTAAAGCAGCTCTGGTCACTCATTTGTACTTTACAATGCCAGTAATA
CTAGTAACAACCACAAAGTAGTCACAAACATAACTAGCAAGAACATTACCCACTTCTATTCTTCTTTCTGTTTGATTCCTTTTCTTTCGGGTGGTTGTTACTCTTTAACCCTATAGCCTTTTA
CGAGTCCTCATAACGGAGGTGGGTTTTATTAAGTTGACAACTAGGCGTAAATAATCAAACCATGAGGATTCCAACTATTATGCATACTAGTTGGTTGGCGATGTCTATATTCAATCATTGTTA
TGCTATATGCATACTAGTTAGTATGTAAACATAAAAAAAGTACTTGTTCAACTTAGTCCCAAACAAGTTAAACTCGGGGTATATGAATATTTACTGACCATATTTCCCGTTTGAATTTATCTC
TTTCGGGATAACAATAACAGTGAAAAGTGTAATTAGACCAAAACTGAAAAGGGTTGTAGTGTCTGAACCTACAAAAAAAAACTCTACACACTCTCTCTTTCTCATGCCAAAAATGAGTCCTTC
CAAGACACCCCAAGTACATTGTGATCAATTAGGAAATGCCCAACAAACTACAACAAGAAGATTCCCACAACCAAGAAACGTTTCTGTTGTGAGGAGCGCACCTATATAAACCTATTCTCTGCC
TACAAAAAAAAAAACCCAGTGCAGTACTGCCTCTTCTTTTAGCTGCACTCAACATATTAATCATTAGCACCAATACTCTCCATCTATAGATTTCCTTTCTAATTTAGGAATTCATTTTAACAT
ATTTATAACAATATTCTCTTTTAAATGGTAGTAATTAGGTGTGCTAAA

Figure S2. The promoter sequence of *SlDYT1*.

The nucleotide in red represents the SlHB8 binding site.

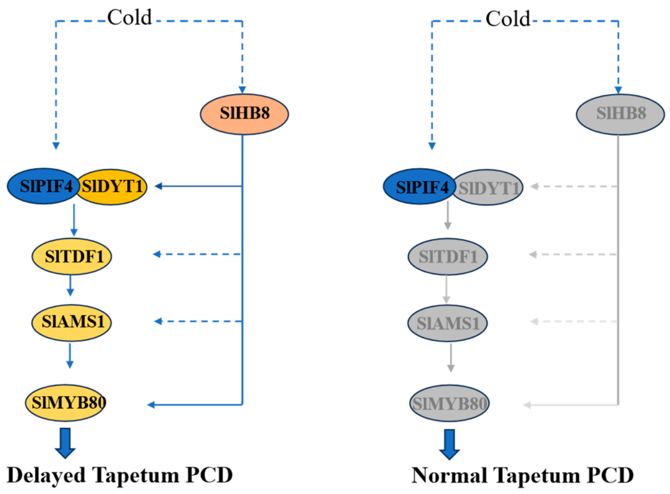

Figure S3. The working model of SlHB8 in regulating tapetum PCD under cold conditions.

Under cold treatment, in wild-type anthers, the increased *SlHB8* expression induces the *SlDYT1* transcripts, which forms a dimer with SlPIF4. This dimer activates the downstream regulators *SlTDF1*, *SlAMS*, and *SlMYB80*, thereby leading to a delay in tapetum degradation and subsequent pollen abortion; however, in *slhb8* mutants, cold exposure does not result in an increase in *SlDYT1* expression, leading to an unsuccessful increase of *SlTDF1*, *SlAMS*, and *SlMYB80*. This lack of increase inhibited the delayed tapetal PCD under cold stress.

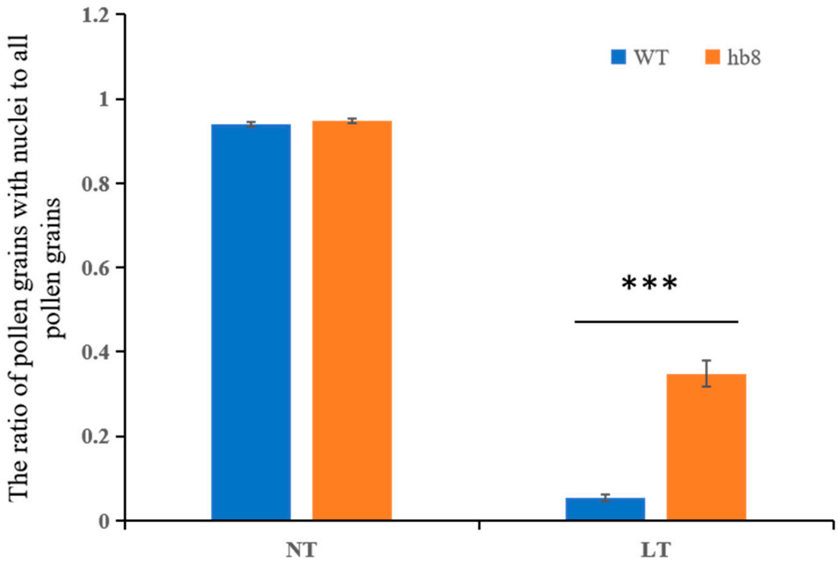

Figure S4. The ration of pollen grains with nuclei to all pollen grains.
NT: normal temperature; LT: low temperature; *hb8*: SlHB8-cr3; The error bars denote SE; *** P < 0.001 (Student's t-test; compared with the WT)
